# DNA profiling and assessment of genetic diversity of relict species *Allium altaicum* Pall. on the territory of Altai

**DOI:** 10.7717/peerj.10674

**Published:** 2021-01-08

**Authors:** Oxana Khapilina, Olesya Raiser, Alevtina Danilova, Vladislav Shevtsov, Ainur Turzhanova, Ruslan Kalendar

**Affiliations:** 1 National Center for Biotechnology, Nur-Sultan, Kazakhstan; 2 Altai Botanical Garden, Ridder, Kazakhstan; 3 Department of Agricultural Sciences, University of Helsinki, Helsinki, Finland; 4 National Laboratory Astana, Nazarbayev University, Nur-Sultan, Aqmola, Kazakhstan

**Keywords:** Relict species, *Allium altaicum*, Molecular marker, Genetic diversity, Interspersed elements

## Abstract

Analysis of the genetic diversity of natural populations of threatened and endangered species of plants is a main aspect of conservation strategy. The endangered species *Allium altaicum* is a relict plant of the Ice Age and natural populations are located in extreme climatic conditions of Kazakstan’s Altai Mountains. Mobile genetic elements and other interspersed repeats are basic components of a eukaryote genome, which can activate under stress conditions and indirectly promote the survival of an organism against environmental stresses. Detections of chromosomal changes related to recombination processes of mobile genetic elements are performed by various PCR methods. These methods are based on interspersed repeat sequences and are an effective tool for research of biological diversity of plants and their variability. In our research, we used conservative sequences of tRNA primer binding sites (PBS) when initializing the retrotransposon replication as PCR primers to research the genetic diversity of 12 natural populations of *A. altaicum* found in various ecogeographic conditions of the Kazakhstani Altai. High efficiency of the PBS amplification method used was observed already at the intrapopulation level. Unique amplicons representative of a certain population were found at the intrapopulation level. Analysis of molecular dispersion revealed that the biodiversity of populations of mountainous and lowland *A. altaicum* is due to intrapopulation differences for climatic zones of habitation. This is likely conditional upon predominance of vegetative reproduction over seed reproduction in some populations. In the case of vegetative reproduction, somatic recombination related to the activity of mobile genetic elements are preserved in subsequent generations. This leads to an increase of intrapopulation genetic diversity. Thus, high genetic diversity was observed in populations such as *A. altaicum* located in the territory of the Kalbinskii Altai, whereas the minimum diversity was observed in the populations of the Leninororsk ecogeographic group. Distinctions between these populations were also identified depending on the areas of their distribution. Low-land and mid-mountain living environments are characterized by a great variety of shapes and plasticity. This work allowed us to obtain new genetic data on the structure of *A. altaicum* populations on the territory of the Kazakhstan Altai for the subsequent development of preservation and reproduction strategies for this relict species.

## Introduction

Currently, monitoring and preservation of biological diversity is one of the most important challenges of the contemporary world. Genetic resources and rare populations and their ecosystems form the basis of biological diversity. A change in species and genetic diversity leads to changes in the ecological system, which affects the stability and preservation of any natural ecosystem. Rare relict and endangered plant species with less genetic potential are more susceptible to the threat of extinction due to changes in environmental conditions and impacts of anthropogenic factors (Edwards et al., 2020; Wessler, 1996). Continuous monitoring of the genetic potential of rare and endangered plant species is necessary. These measures should include the analysis of intrapopulation polymorphisms, genetic differentiation of populations, and studying species morphology, biological signatures of the population (e.g., age composition of the population), and cross-species communities (Morris et al., 2002). The application of currently existing molecular genetic research methods is very important when choosing a strategy for the conservation of rare relict and endangered plant species, since these methods make it possible to reveal the genetic structure of a species and the genetic diversity in populations and between them. They also allow the development of measures for reduction of genetic drift for protected plant species through environmental monitoring, and, if necessary, measures to preserve rare genotypes (Altukhov, 2006; Ryabushkina et al., 2008; Vincent et al., 2020).

Representatives of the *Allium* L. genus are perennial herbaceous plants belonging to the *Alliaceae* subfamily, the *Amaryllidaceae* family. Substantially, all onion species are valuable food, medical, or adornment plants and are traditionally preserved by human beings, which leads to significant depletion of their reserves in nature. The *Allium* L. genus is represented by about 750–800 species. It is one of the largest genera of the Kazakhstani flora, which includes 140 species, 21 species of which occur in the territory of the Kazakhstani Altai, the Saur Manrak, and the Zaisan basin (Abugalieva et al., 2017; Allkin, Lughadha & Paton, 2013). Some representatives of the *Allium* genus are listed in the Red Book of several states such as Mongolia, Russia, and China due to their high biological value (National Reports & NBSAPs, 2006; Dimeyeva et al., 2015).

Among onions, there are several numerically insignificant species that are of scientific interest in terms of studying their biodiversity and thus also need additional conservation measures. One of them is a relic of the Ice Age *Allium altaicum*, ubiquitously reducing its number due to mass collection for consumption as food. Uncontrolled mass harvesting destroys mother plants, which significantly reduces seed renewal and leads to a decrease in the number of populations and distribution areas. Currently, *A. altaicum* plants occur in the territory of the Kazakhstani Altai only on almost inaccessible mountain slopes. Some populations are sporadic and sometimes they can be represented by several specimens (Dimeyeva et al., 2015). The strategy for conservation and reproduction is a very urgent task in the conservation of biodiversity of the natural flora of Kazakhstan and must addressed using modern approaches (Ivachenko et al., 2006). Contemporary molecular genetic analysis methods yield more precise and objective data on the genetic structure of populations, which can reduce the negative impact of anthropogenic factors.

Molecular genetic methods based on polymorphisms of certain genome sequences or proteins are applied to research the genetic polymorphisms of rare plant species. Various *Allium* species were already studied using various molecular genetic PCR markers such as RAPD, ISSR and AFLP (Buso et al., 2008; Chen et al., 2014; Dyachenko, Seredin & Filyushin, 2019; Mitrová, Svoboda & Ovesná, 2016; Sudha et al., 2019; Villano et al., 2018). However, these PCR techniques are not efficient enough to determine genetic diversity or have problems with reproducibility or cost (Nybom, 2004). Using microsatellite markers (SSR) would have been promising if such markers had been developed for all onion species (Barboza et al., 2018; Karic et al., 2018). However, these markers are expensive during initial development and require genome sequencing of several genetically distant genotypes for each species. This approach also requires subsequent comparative bioinformatic analysis of these genomes to search for polymorphic SSR loci and to select effective PCR primer pairs. This is difficult and expensive work and is usually only requested for critical agricultural crops requiring these types of markers for identification of and genotyping selection lines. Sequencing the plastid genome of the bulb onion *Allium cepa* (Filyushin et al., 2018; Huo et al., 2019) does not solve the problems of intraspecific diversity analysis. To study the intraspecific genetic polymorphism of the *Allium* wildlife relict species, it is preferable to use markers characterized by a wide distribution in a genome and, above all, the accessibility for any species including those that have not been studied. Such DNA genetic markers include all PCR variants of the RAPD method, such as ISSR, SCoT, and others. This list can also be complemented by methods that are based on the interspersed repeat sequences in genomes (Belyayev et al., 2019), including a variety of transposable elements, 5S rRNA and tRNA related sequences, regions of some promoters, and introns. Such types include PCR markers based on the use of conserved sequences for various classes of retrotransposons (Kalendar et al., 2010). Here, the fundamental feature of these types of sequences is that in the whole variety of classes of mobile genetic elements and their sequences there are conserved regions for specific classes of mobile genetic elements (transposons, retrotransposons), which can be universally used for plants and animals and for species studied for the first time (Kalendar, Amenov & Daniyarov, 2018).

The genetic polymorphism research method in rare species using retrotransposons is a simple and accessible method as the RAPD method is used (Abdollahi Mandoulakani et al., 2015; Doungous et al., 2015, 2019; Smykal et al., 2009). Variations of these markers have been used in various species of fungi, plants, animals and humans (Doungous et al., 2015; Kalendar & Schulman, 2013; Li et al., 2020; Turzhanova et al., 2020; Vuorinen et al., 2018). Due to the fact that many mobile genetic elements “mix” with each other in the process of intra- and intrachromosomal recombination, this leads to convergence of these conserved regions and makes PCR amplification possible. Such universal regions for retrotransposons include the tRNA priming binding site (PBS) when initializing long-terminal repeat (LTR) retrotransposon replication through reverse transcription of their RNA and integration of the resultant cDNA into another locus (Havecker, Gao & Voytas, 2004). The sequences of the PBS region are complementary to at least 12 nucleotides of the tRNA sequences, which is already sufficient for their use as PCR primers (Kalendar et al., 2010). Since retrotransposon sequences are frequent near each other in reverse orientation, PBS sequences can then be accessible when used for DNA amplification for the majority of eukaryotic species with large genomes, such as plants, animals, and humans (Kalendar, Amenov & Daniyarov, 2018; Kalendar et al., 2010; Schulman & Kalendar, 2005). Primers complementary to interspersed repeats allow amplification of the region between these repeats if the distance between the repeats does not exceed the processivity of the used DNA polymerases (Kalendar et al., 2019). Therefore, this approach, based on PCR between interspersed repeats and mobile genetic elements, can be easily adapted for any eukaryotic species for the rapid detection of molecular genetic polymorphisms even in an agarose gel (Doungous et al., 2019; Hosid et al., 2012; Kalendar, Amenov & Daniyarov, 2018; Kalendar & Schulman, 2013; Milovanov et al., 2019; Turzhanova et al., 2020). Thus, application of PCR methods based on the use of conserved sequences of interspersed repeats and mobile genetic elements allows universal and efficient detection of polymorphisms for almost every eukaryotic species (Kalendar, Amenov & Daniyarov, 2018; Smykal et al., 2009).

The use of these type of markers for rare relict and endemic plant species is supported by the direct participation of some mobile genetic elements in plant adaptation to stress. Multiple changes at all levels of organization, including the chromosomal level, can be genetically fixed during vegetative reproduction under the influence of stressful living conditions (Belyayev et al., 2010; Hosid et al., 2012; Kalendar et al., 2000).

Therefore, under stress conditions, bursts in activity of retrotransposons and transposons can be fixed genetically in plants with vegetative reproduction. The induced genetic rearrangements and insertions of mobile genetic elements in regions of active euchromatin contribute to genome alteration, which leads to “genomic stress” (Belyayev et al., 2010; Capy et al., 2000; Kalendar et al., 2020). Transcriptionally active retrotransposons can potentially participate indirectly in gene regulation and adaptation to environmental stress, since their activity is induced by stressful environmental conditions (Belyayev et al., 2010; Capy et al., 2000; Vicient et al., 2001; Wessler, 1996). As a result, these chromosomal changes under the influence of mobile genetic elements can potentially increase the adaptive potential of specimens to stress conditions (Belyayev et al., 2010; Capy et al., 2000; Grandbastien, 1998; Horvath, Merenciano & Gonzalez, 2017; Voronova et al., 2020).

The climatic conditions of the *A. altaicum* relict onion species in the territory of the Kazakhstani Altai are extreme, characterized by wide fluctuations of temperature within 24 hours and a high insulation. This leads to the appearance of various morphobiotypes within the population due to the variability of many traits that determine genetic polymorphism (Ivachenko et al., 2006).

This study examined the genetic diversity of the *A. altaicum* relict species that inhabit various ecological and geographical regions of Altai using DNA profiling based on conserved PBS sequences of interspersed repeats of retrotransposons. There is a significant morphological diversity of *A. altaicum* forms in the mountainous and lowland Kazakhstan Altai. In some populations, the seed form of reproduction also prevails and in others the vegetative form prevails. In addition, the populations are represented by individuals in different age states. These studies are of evolutionary interest, since separation of the *A. altaicum* forms in the territory of the mountainous and lowland Kazakhstani Altai occurred within the long period; in some populations, the seed form of reproduction primarily prevails while in others it is vegetative.

We assume that the genetic and morphological diversity of the Altai *A. altaicum* populations that inhabit the very contract climatic conditions of the Kazakhstan Altai may be associated with the activity of mobile elements. As a result, the observed genetic and morphological changes contribute to an increase in the ecological plasticity of the species.

## Materials and Methods

### Plant material

**Figure 1:**
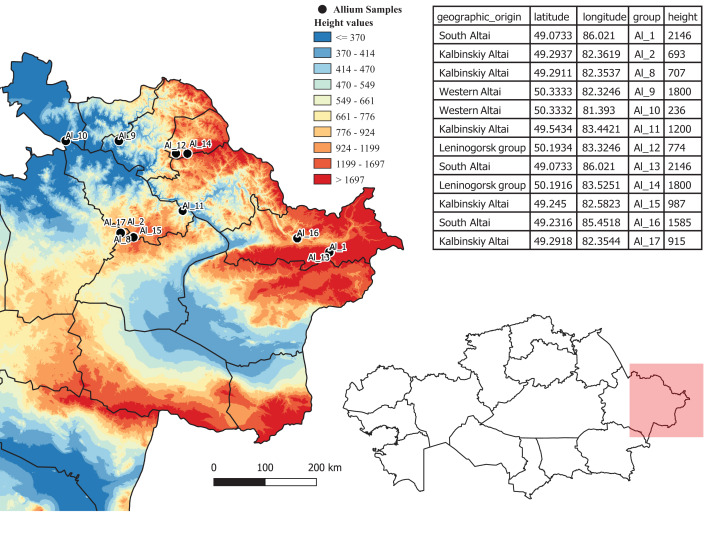
Places of collection of samples of rare and endemic species of onions in the territory of Kazakhstani Altai.

**Figure 2:**
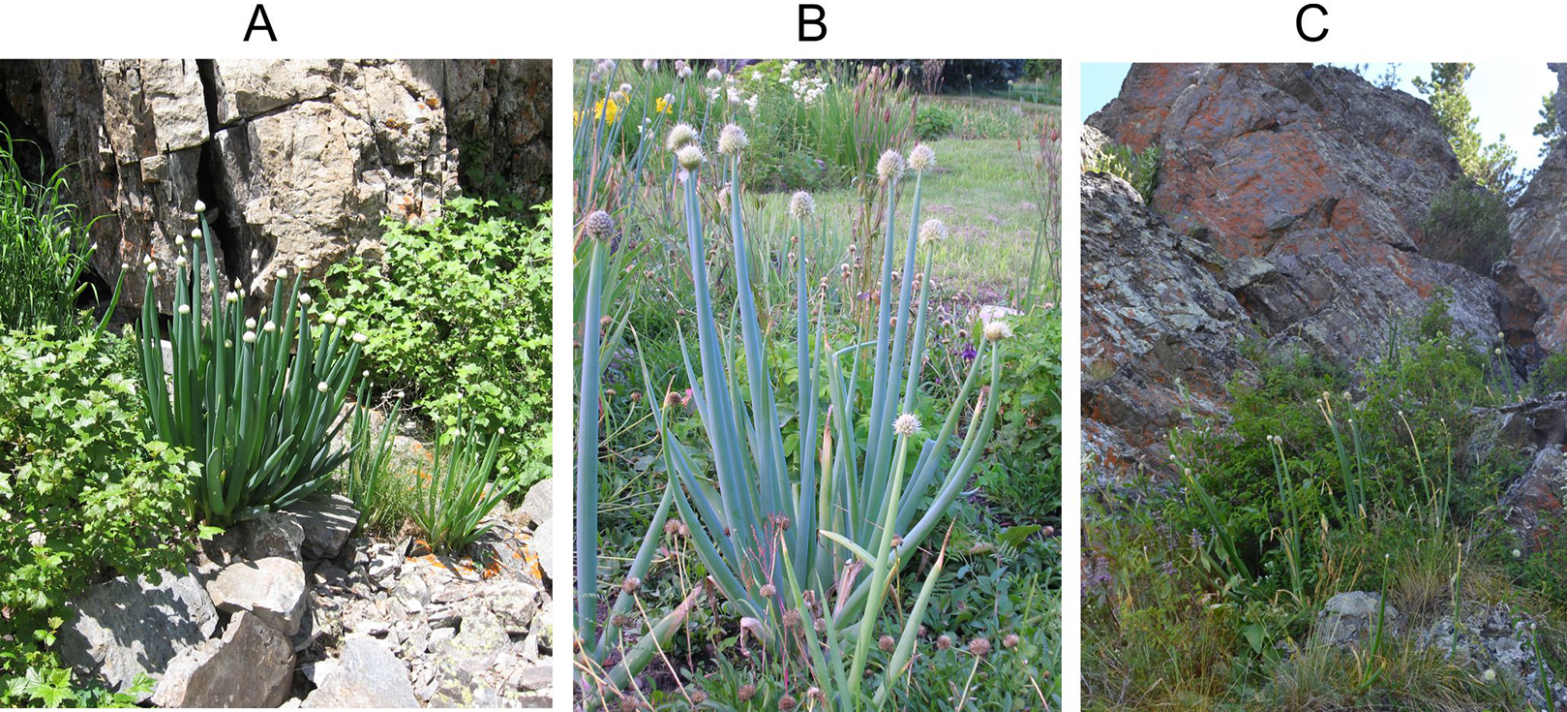
Plants of *A.altaicum*. (A) *A. altaicum* population in the Seriy Lug Ecosite; (В) *A. altaicum* population (blue morphotype) in the Sibir Lakes vicinities in the territory of Kalbinskii Altai; (С) *A. altaicum* population on the South Altai Tarbagatai range, Burkhat passage.

Ten samples for every 12 populations of the endangered onion *A. altaicum* were collected in places of their natural vegetation in the territory of mountainous and lowland Kazakhstani Altai and were for this study (Fig. 1). Sample collection depended on temperature conditions and melting of snow cover. To study morphological traits, at least 10 plants were used for high-mountain populations and 10–18 plants for the remaining populations. DNA was isolated from biological material (seeds, fresh or dry leaves) from at least 10 *A. altaicum* plants. The positions and absolute height of the coenopopulation location from which the plant material was taken were determined using a Garmin GPS 72H GPS navigator (Fig. 1). The population habitat is shown in Fig. 2.

### DNA isolation and amplification

Genomic DNA was extracted from fresh plant leaves using modified acid CTAB extraction buffer (2% CTAB, 2 M NaCl, 10 mM Na_3_EDTA, 100 mM HEPES, pH 5.3) with RNAse A treatment (http://primerdigital.com/dna.html) (Kalendar, Boronnikova & Seppänen, 2021).

Universal PBS primers (Kalendar et al., 2010) were used to assess the genetic diversity of various *A. altaicum* populations. Nucleotide sequences of these PBS sequences are universal for all LTR retrotransposons and belong to highly repeated repeats typical for higher eukaryotes (Phong et al., 2016). The length of PBS sequences of retrotransposons at which the primers were oriented in both directions did not exceed 18 nucleotides. Sequences of the used PBS primers are represented in Table 1 (Kalendar et al., 2010).

**Table 1:** Sequences of 18-mer PBS primers used in this study and comparative analysis of products of iPBS amplification of the DNA of *Allium altaicum* populations.

ID	Sequence 5′–3′	Annealing temperature (°C)	Number of amplified amplicons	Number of polymorphic amplicons	Total polymorphism level, %	PIC	Shannon information index
2221	ACCTAGCTCACGATGCCA	56.9	39	35	90	0.719	0.310
2237	CCCCTACCTGGCGTGCCA	55.0	23	21	91	0.794	0.286
2240	AACCTGGCTCAGATGCCA	55.0	25	23	92	0.779	0.290
2249	AACCGACCTCTGATACCA	51.0	23	17	74	0.746	0.309
2257	CTCTCAATGAAAGCACCA	50.0	15	15	100	0.689	0.295
2373	GAACTTGCTCCGATGCCA	51.0	20	19	95	0.831	0.271
2395	TCCCCAGCGGAGTCGCCA	52.8	20	20	100	0.769	0.286
Total			165	150			

The genetic variability of *A. altaicum* samples was analyzed using PBS primers designed in (Kalendar et al., 2010). PCR reactions were performed in a 25-µl reaction mixture. Each reaction mixture contained 25 ng template DNA, 1× Phire^®^ Hot Start II PCR buffer with 1.5 mM MgCl_2_, 1 µM primer, 0.2 mM each dNTP, and 0.2 µl Phire^®^ Hot Start II DNA polymerase (Thermo Fisher Scientific Inc., Waltham, MA, USA). PCR amplification was performed in a Bio-Rad Thermal Cycler T100 under the following conditions: initial denaturation step at 98 °C for 1 min; 30 cycles at 98 °C for 5 s at 57 °C (depending on primer sequence) (Kalendar et al., 2017; Kalendar, Lee & Schulman, 2011) for 20 s; 72 °C for 60 s; final extension at 72 °C for 2 min. All PCRs were repeated at least twice for each isolate. All PBS primers were tested to assess the genetic diversity of *A. altaicum* using iPBS amplification for DNA profiling. Primers that generated few PCR products were excluded. Primers with a weak profile or that produced mainly monomorphic amplification products were also excluded. PCR products were separated by electrophoresis at 70 V for 8 h in a 1.2% agarose gel with 1× TBE buffer. A Thermo Scientific (100–10,000 base pairs) GeneRuler DNA Ladder Mix (#SM0332) was used as a standard DNA ladder. The PCR products were visualized with a ChemiDoc-It2 Imaging System (UVP, LLC, Upland, CA, USA; Analytik Jena AG, Jena, Germany) and a PharosFX Plus Imaging System (Bio-Rad Laboratories Inc., Hercules, CA, USA) with a resolution of 50 µm after staining with ethidium bromide. PBS primers generated in the PCR yielded clearly distinct amplification products, showing considerable variability among the isolates belonging to different populations of *A. altaicum*.

### Data scoring and analysis

Only clear scorable bands were used for studying genetic variability among the isolates of *A. altaicum*. Each band of a unique size was assumed to correspond to a unique locus (Table 1). To construct a binary matrix, reproducible fragments were scored as present (1) or absent (0). GenAlex 6.5 (Peakall & Smouse, 2012) was used to calculate the total number of alleles, Shannon information index (I), genetic differentiation index (PhiPT) among populations, and the number of private alleles per population (Supplemental Data 1 and 2). Analysis of molecular variance (AMOVA) among and within populations was also calculated with GenAlex 6.5 computer software. PCoA was performed to supplement the findings obtained from cluster analysis using the GenAlex 6.5. Each principal component can be characterized graphically by a score plot that shows how samples are related to each other (Kumar et al., 2018).

## Results

### *Allium altaicum* sample collection and characteristics

*Allium altaicum* (2*n* = 16) is a rare plant and a glacial relict. In Kazakhstan, *A. altaicum* grows in the territory of the Kazakhstani Altai in the following geographical regions: Western Altai (Ivanovsky, Ulbinsky, Ubinsky, Listvyaga ranges); Southern Altai (Southern Altai, Tarbagatai, Narymsky, Sarymsakty ranges); Kalbinskii Altai (Kalba range) and on the Ukok plateau (Abugalieva et al., 2017; Dimeyeva et al., 2015; Kamenetsky & Rabinowitch, 2006). *A. altaicum* populations were found at altitudes from 693 m to 2,146 m above sea level. In the territory of the south-west periphery of the Western and South Altai, there *A. altaicum* were distinguished by several groups of coenopopulations of this species.

Geographic data on their location, altitude of habitat, and population characteristics are shown in Table S1. We believe that almost the entire *A. altaicum* gene pool in the territory of the Kazakhstani Altai was obtained.

Phenotypic variation analysis showed that the greatest differences were found in the number of generative shoots, number of flowers in the inflorescence, mass of bulbs, and number in the seat. Thus, the number of generative shoots varied from minimum values of 1–5 in the plants from Al1 and Al13 populations to 7–11 in the plants from Al11 and Al13 populations. The minimum value of bulb mass (3.3 g) was observed in Al13 population located at an altitude of 2,146 m above sea level, while maximum values (25.1 g) were observed in plants from the Al9 population.

The analysis of indicators depending on the altitude of the populations’habitat shows that in the populations located in the mid-mountain regions (1,000–2,000 m above sea level), the maximum values of productivity of the generative organs were observed. Lowland and high-mountain populations were distinguished by lower productivity with the exception of the Al12 population, which is located in milder climatic conditions in the territory of the Altai Botanical Garden (Ridder town). The polymorphism of phenotypic traits in the studied populations is clearer when differentiated by altitude of distribution range than by geographic location.

### iPBS loci variability analysis

DNA amplification of *A. altaicum* revealed clearly distinguishable amplicons whose number varied depending on the primer used. The level of the detected polymorphism and saturation of the amplification range were the efficiency criteria for the primers used. 165 PCR fragments were obtained in total, of which 150 were polymorphic between the studied samples (Table 1).

**Figure 3:**
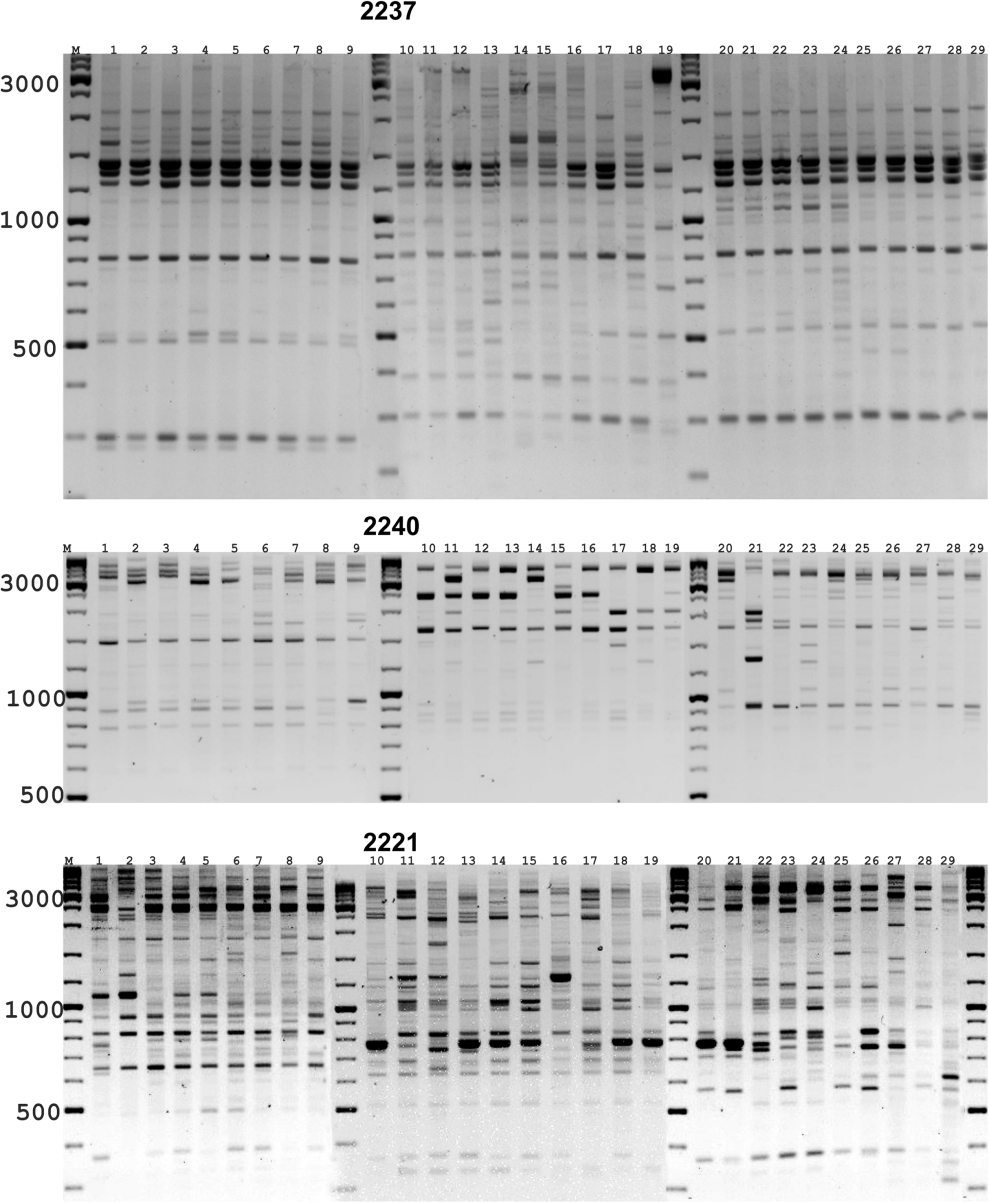
Analysis of individual seeds from *Allium altaicum* populations using the PBS primers 2237, 2240 and 2221. Sample order: *Al*1 (1–9), *Al*11 (10–19), *Al*17 (20–29). Distribution of *Allium* population above the sea level: *Al*1—2,146 m, *Al*11—1,200 m, *Al*17—915 m. M—Thermo Scientific GeneRuler DNA Ladder Mix (100–10,000 bp).

Depending on the primer, the number of informative amplifiable fragments varied from 15 to 39 and their size varied from 500 to 3,500 bps. The polymorphism level varied from 74% to 100% and was sufficient for differentiation of the studied genotypes. The studied samples were distinguished by the individual bands of polymorphic amplified fragments of various molecular weight (Fig. 3). Based on DNA fingerprint data, the main indicators of genetic polymorphism were calculated reflecting the variability level in the studied populations of *A. altaicum*. Evaluating the resolution of PBS primers in the study of polymorphism in *A. altaicum* populations, polymorphism indices (PIC) varied from 0.689 for primer 2,257–0.831 for primer 2,373, with an average value of 0.761. All studied primers had PIC values above 0.5, which indicates the efficiency of their use when studying the genetic polymorphisms of *Allium altaicum*. The minimum values of the Shannon biodiversity index (I) were obtained when using primer 2,373 and the maximum values when using primers 2,221 and 2,249.

### Genetic diversity of Kazakhstani populations of *A. altaicum*

**Figure 4:**
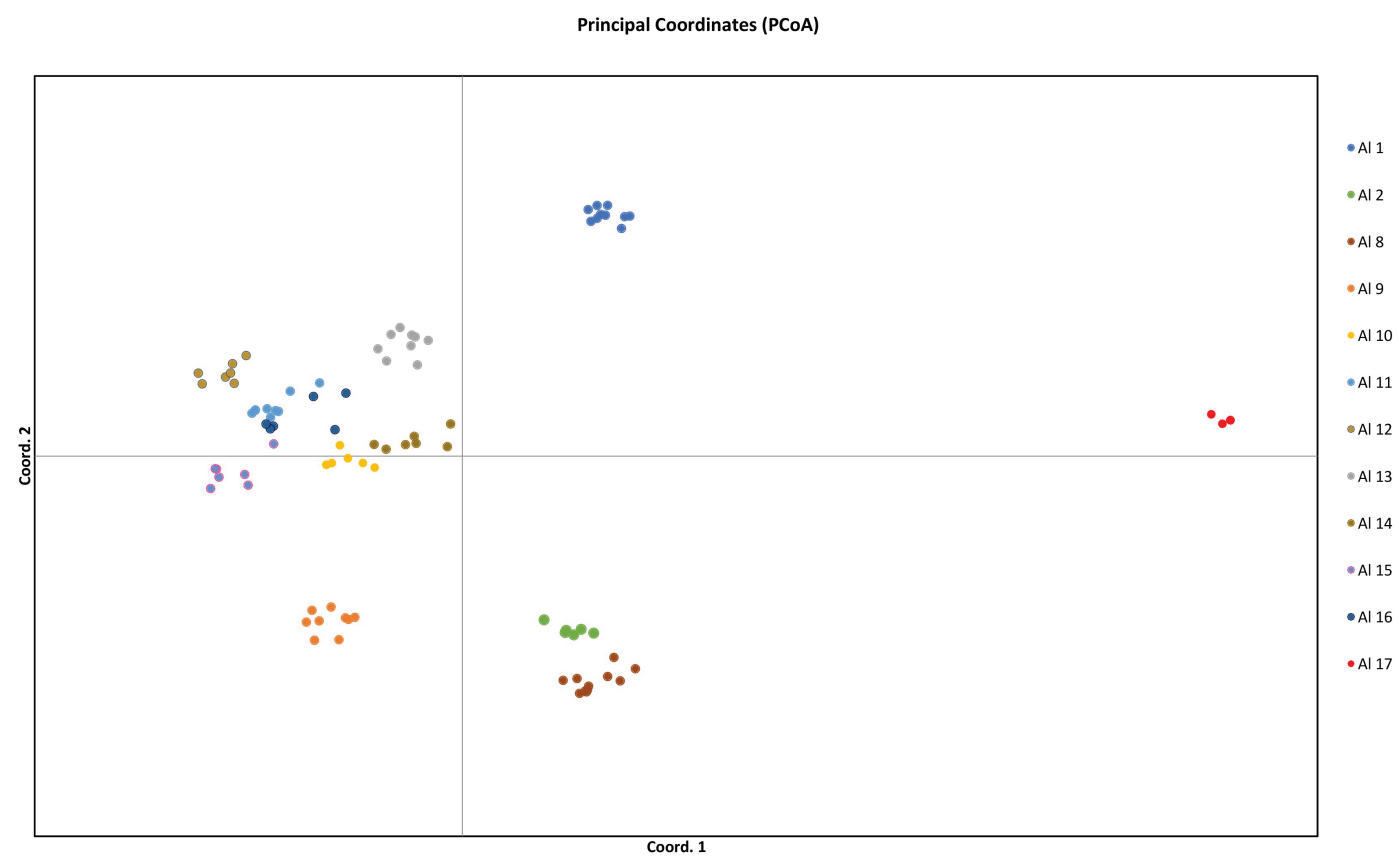
Principal coordinates analysis (PCoA) based on Nei’s genetic distances, reflecting the diversity of *Allium altaicum* populations located in the territory of Kazakhstani Altai, based on the results of DNA profiling and assessment by using PBS.

The data obtained from amplification with PBS primers of various populations of *A. altaicum* were used for GenAlex 6.5 genetic analysis and for generating the PCoA ordination diagram (Fig. 4).

We previously divided the *A. altaicum* populations into groups based on their geographic location in the territory of the Kazakhstani Altai based on the geographic coordinates of the populations determined using a GPS navigator (Fig. 1; Table S1). In total, four geographically isolated locations with different environmental conditions were identified (Table 2).

**Table 2:** Distribution of populations of *Allium altaicum* by geographic habitat and their characteristics.

Group of populations	Absolute temperature maximum/minimum, °C	Average annual temperature, °C	Amount of precipitation during the warm period (April–October), mm	Soil covering	Population characteristics
South Altai: Al1 Al16 Al13	36°/−44°	5.1	350–550	High content of humus, permanent soil moistening from moderate to abundant. The ground cover is well developed, composed of litter, often with a thick layer of ground mosses and lichens.	Populations in the form of individual spots with the area of 150–300 m^2^. Populations are full-grown, normally aging, with high rates of seed productivity.
West Altai: Al9 Al10	41°/−43°	3.1	280–400	Low humus content, lack of moisture. The ground cover is poorly expressed	Populations occur sporadically, as individual spots 100–200 m^2^. Populations are full-grown, with a predominance of pregenerative individuals. Seed reproduction is weak, with low plant preservation in the early stages of development due to lack of moisture in the spring and summer and severe winter climatic parameters
Kalbinskii Altai: Al2 Al8 Al11 Al15 Al17	42°/−48°	−2.1	182–250	The soil layer is not expressed, there is a strong moisture deficit. Above soil cover is formed from needles, cones, litter and decomposition products of granitoids.	The populations are limited in the area of settlement, are of an insular nature (2–3 m^2^). Seed regeneration is satisfactory. Populations and full grown, progressive with the domination of generative individuals of different ages.
Leninogorsk: Al12 Al14	40°/−44°	3.2	289–435	The soil covering is with low humus content, the ground cover is poorly expressed by larch and coniferous litter.	The populations are very sparse, form small arrays with a low density (3–15 individuals per 10 m^2^). Seed regeneration is satisfactory. Populations and full-grown, with normal aging, progressive with the domination of generative individuals of different ages.

The values of the main induces of genetic polymorphism determined based on the analysis of 165 loci for each of 12 populations of *A. altaicum* included in the analysis are shown in Table 3.

**Table 3:** Summary of *Allium altaicum* population geographic diversity indices calculated on the basis of iPBS markers.

Group	N	PPL	Na	Ne	I	He	uHe*
South Altay	3	35.76	0.921	1.239	0.204	0.138	0.166
Leninogorsk	2	12.73	0.570	1.090	0.077	0.053	0.070
Kalbinskii Altay	5	63.09	1.509	1.378	0.346	0.227	0.252
West Altay	2	16.36	0.655	1.116	0.099	0.068	0.090
Average value	3	33.48	0.914	1.206	0.181	0.121	0.145

**Note:**

* PPL, percentage of polymorphic loci; Ne, number of effective alleles; Na, number of different alleles; I, Shannon’s information index; He, expected heterozygosity; uHe, unbiased expected.

These results suggest that natural populations of *A. altaicum* from the Kalbinskii Altai were characterized by high indices of genetic polymorphism in comparison with other populations. Thus, the number of polymorphous loci amounted to 63%, which is twice more than the average value for all populations. This index for the Leninogorsk group was almost five times lower and was almost four times lower in populations from the western Altai. The average number of alleles per locus (Na) varied from 0.570 in the Leninogorsk populations to 1.509 in populations from the Kalbinskii Altai. The average number of effective alleles varied from 1.090 to 1.378. The analysis of genetic structure of *A. altaicum* populations showed that between the distinguished groups of populations, there were substantial differences regarding the level of heterozygosity; the maximum values were observed in populations from the Kalbinskii Altai and the minimum values were observed in the Leninogorsk populations. The Shannon biodiversity index (I) showed that the average minimum length of a unique binary taxonomic code of one structural unit from the analyzed collection or panel varied from 0.077 (Leninogorsk group of populations) to 0.346 (populations from the Kalbinskii Altai).

The AMOVA analysis of the frequency variation of PBS amplicons for 12 populations of *A. altaicum* showed that the differences were more pronounced at the interpopulation level (89%, d.f. = 11%) (Table 4). The data were analyzed using Fisher’s exact test and *P*-values of ≤ .05 were considered to be statistically significant. The *P*-value from Fisher’s test showed significant differences between groups (*P* < 0.0001). The PhiST differentiation index (similar to the Fst and Gst indices) had a value of 0.88, which was significant and indicated a high differentiation between populations (89% of the total genetic variability is due to differentiation between populations).

**Table 4:** Results of molecular dispersion analysis (AMOVA) between local groups of *Allium altaicum* populations.

Variability type	Df	SS	MS	Est. Var.	%	PhiPT	P*
Among Populations	11	1,711	155.5	19.2	89%	0.887	0.001
Within Populations	84	206	2.5	2.5	11%		
Total	95	1,916		21.7			

**Note:**

* Df, degree of freedom; SS, sum of squares; MS, mean square; Est. Var., estimated variance; PhiPT, genetic differentiation index among population.

The analysis of the spatial dynamics of *A. altaicum* populations performed using the multivariate ordination method and the principal coordinates analysis (PCoA) based on NeiP data showed that only a single compact group was observed, which was formed by the Al10, Al11, Al12, Al13, Al14, Al15, and Al16 populations (Fig. 4). Such a compact arrangement of populations with respect to the PCoA coordinate axes is typical in the case of close genetic similarity.

The remaining five populations of *A. altaicum* were located remotely on different parts of the diagram. Thus, the Al9 population located on the south-eastern slope of the Porozhnaya mountain (Ubinskiy range) is separate from the compact group relative to the PCoA2 coordinate axis. The Al1, Al8 and Al2 populations were also located separately from the general group in the PCoA1 coordinate. The Al17 population, one of the highest mountain populations, was located completely apart from other populations relative to both axes of coordinates. The PBS loci analysis demonstrated the absence of any geographic reference to the geographical areas identified by us, and populations geographically distant from each other were often located nearby.

We also analyzed the genetic diversity of *A. altaicum* depending on the altitude of their habitat. For this purpose, the populations were divided into three groups. Low populations in the form of larch park forests contained six populations (Al2, Al8, Al10, Al12, Al15, Al17). Four populations were included in the group of mid-mountain populations of *A. altaicum* located at an altitude of 1,000–2,000 m above sea level, where the vegetation is represented by light coniferous taiga and colorful Alpine meadows (Al9, Al11, Al14, Al16). Only two populations of *A. altaicum* were included in the high-mountain group located at over 2,000 m above sea level in the zone of tundra vegetation (Al1 and Al13) (data from Table 1).

The most significant differences were found between high-mountain and mid-mountain populations. The level of genetic differences consisting of the number of PPL polymorphous loci was four times lower in high-mountain populations. The number of observed alleles (Na) in mid-mountain populations was two times higher, and indicators such as the observed heterozygosity (He) and Shannon biodiversity index I were over three times higher (Table 5).

**Table 5:** Biodiversity of *Allium altaicum* populations based on altitude of the area of distribution above sea level.

Pop	Altitude above sea level	PPL	N	Na	Ne	I	He	uHe*
Low mountain	1,000 m	44.24	6	1.061	1.267	0.231	0.155	0.169
Mid-mountain	1,000–2000 m	64.85	4	1.430	1.378	0.341	0.226	0.258
High mountain	2,000 m	16.36	2	0.655	1.116	0.099	0.068	0.090
Total		41.82	4	1.048	1.254	0.224	0.149	0.172

**Note:**

* PPL, percentage of polymorphic loci; Ne, number of effective alleles; Na, number of different alleles; I, Shannon’s information index; He, expected heterozygosity; uHe, unbiased expected.

Analysis of variance of populations depending on the height of the range showed that all differences are at the intrapopulation level (96%, D.f. = 9) (Table 6).

**Table 6:** Results of molecular dispersion analysis (AMOVA) between *Allium altaicum* populations from a different altitude above sea.

Source	Df	SS	MS	Est. Var.	%	PhiPT	*P*
Among Populations	2	46.333	23.167	0.778	4%	0.037	0.192
Within Populations	9	182.833	20.315	20.315	96%		
Total	11	229.167		21.093	100%		

## Discussion

Rare relict species of plants undergoing super-exploitation are under threat of ecological and biological extinction. Relict plant species are of great scientific value as a reliable source of information on the vegetation cover of past geological eras. To preserve the biodiversity of rare plant species and to reduce the negative influence of anthropogenic factors, it is necessary to thoroughly research natural populations at the molecular genetic level. This allows analysis of the population structure for following selection of relict gene pool preservation and reproduction structure. Currently, in the era of a sharp increase in anthropogenic stress and the threat of ecological catastrophe, the preservation of relict flora is urgent.

Studies on the genetic diversity of *Allium* were mostly conducted on the onion species related to human food and pharmacology and are actively applied in the sphere of selection (Chen et al., 2014; Morales et al., 2013). For studying the biodiversity of unexplored, wild species and minimally studied taxonomic groups, such as *A. altaicum*, simple and universal PCR methods, such as the RAPD method, are most promising (Friesen et al., 1999; Shigyo, Miyazaki & Tashiro, 2002). The simplicity of the RAPD method and its low cost leads to problems of low reproducibility for some primers in interlaboratory studies. These problems are associated with the sensitivity of the RAPD method to the PCR conditions and the quality of the DNA being used. Next generation sequencing methods cannot replace any of the existing approaches based on PCR with one or a pair of primers (RAPD, ISSR and others) (Friesen, Fritsch & Blattner, 2006). These methods are applicable to any organism, including prokaryotes. One of the variants of such PCR methods is an approach based on the use of conserved sequences of interspersed repeats and mobile genetic elements (Kalendar, Amenov & Daniyarov, 2018).

These types of PCR markers are of interest because a plant genome can contain up to 90% of repetitive DNA sequences, more than a half of which are represented by retrotransposons (Doungous et al., 2015; Kalendar & Schulman, 2013). Due to the fact that retrotransposons are widespread within the whole genome, they participate in recombination during meiosis and mitosis, lead to the genome instability via inversions or translocations, and are widely used as molecular markers. Specifically, PCR methods based on detection of transposable element insertion site polymorphisms include Inter-retrotransposon amplified polymorphism (IRAP) (Kalendar et al., 1999) REtrotransposon-Microsatellite Amplified Polymorphism (REMAP) (Kalendar & Schulman, 2006, 2013), Sequence-Specific Amplification Polymorphism (SSAP) (Waugh et al., 1997), and the Inter-primer Binding Site (iPBS) amplification technique (Kalendar & Schulman, 2013).

Improved design of PCR primers from interspersed repeats sequences make these approaches more efficient than any variants of the RAPD and ISSR methods. Reproducibility of PCR and high polymorphism can be used as an express technology in assessing the ecogeographic diversity of any plant species (Kumar et al., 2018; Phong et al., 2016). Retrotransposons are activated in response to the influence of various forms of abiotic and biotic stresses, and this can lead to genome destabilization (Belyayev et al., 2010; Kalendar et al., 2020; Vicient et al., 2001; Wessler, 1996). Retrotransposons can be one of the sources of genetic variability induced by the appearance of adaptation traits due to accidental insertions/deletions of retrotransposons. Retrotransposons enhance the likelihood of mutations and recombination, which allow the organism to exist in extreme conditions (Galindo-Gonzalez et al., 2017). Molecular markers based on interspersed repeats have been efficiently used on rare or endemic plant species whose genomes have not been studied (Kalendar & Schulman, 2013; Kalendar et al., 2019; Milovanov et al., 2019) and have also been widely used on a wide range of plants (both monocotyledons and dicotyledonous (Abdollahi Mandoulakani et al., 2015; Nie et al., 2019; Vuorinen et al., 2018; Woodrow et al., 2010; Yilmaz et al., 2018)) and in animals and fungi (Turzhanova et al., 2020).

Studies on the genetic diversity of the rare species *Allium altaicum* using markers based on conserved PBS sequences in retrotransposons have not been previously performed. This relict species of onions grows in the hot climatic conditions of the Kazakhstani Altai. The main environmental factors that determine its life cycle are the severe and poorly favorable meteorological conditions of the growing season (daily drops in air temperatures down to negative values; intense solar radiation; strong and constant cold winds; summer snowfalls; freezing; snow drifts in the winter; bare soil; thin, coarsely skeletal, and extremely nutrient-poor soil substrate; powerfully developed above-ground cover, which prevents soil heating) (Ivachenko et al., 2006). Some populations have a weak ability to form daughter bulbs and low seed productivity. Seed sprouting is lengthy and can take several years. All of these factors limit wide distribution along with massive uncontrolled collection by the population (Dimeyeva et al., 2015; Kamenetsky et al., 2004).

Studies using the PCR method with PBS primers revealed high-resolution detection of the genetic diversity level of wild-growing populations of *A. altaicum*. The polymorphism index (PIC) is an important indicator that evaluates the efficiency of polymorphic loci and determines the efficiency of each primer. Under the classification of Botstein et al. (1980), highly informative primers are those with a PIC ≥ 05. The average informative primers are those with a PIC value between 0.5 and 0.25; low informative primers are those with a PIC ≤ 0.25. In our studies, PIC varied from 0.689 to 0.831 with an average value of 0.761, which indicates their high information content and the efficiency of their use in the study of genetic polymorphism of the *Allium* genus. There are studies on the analysis of the genetic diversity of onions (*A. cepa* L.) using RAPD- and ISSR-specific markers. In these studies, the PIC values were much lower and were within the limits of 0.07–0.5 for the studied genotypes for the RAPD markers. The PIC values were between 0.06 and 0.26 for the ISSR markers for the same genotypes (Sudha et al., 2019).

In our studies, the total level of genetic diversity of *Allium altaicum* populations was low, which may be associated with the restricted distribution area of this species and the effects of genetic drift (Broadhurst et al., 2017; Nybom, 2004). Oftentimes, endemic and relict species have a low level of the expected heterozygosity (*uHe*), the average meaning of which does not exceed 0.2. In our studies, this level of heterozygosity was only in *Allium altaicum* populations included in the Kalba local ecogeographic group. The substantial variety of populations of this group was conditioned by the dominance of multiple-aged generative specimens in their composition, notwithstanding the extreme conditions. The ability to adapt to severe conditions was demonstrated by the frozen tops of leaves and buds during the collection period. Furthermore, populations belonging to this ecogeographic group were located in hard-to-reach locations. This restricts mass collection by the population and allows the plants to form full seeds in favorable years. This points to the existence of seed and vegetative reproduction in these populations.

The low levels of population diversity revealed in the populations of the Leninogorsk and West Altai local groups can be explained by the influence of anthropogenic factors (the proximity of settlements, collection by the population at certain periods of the growing season). Due to the collection by the population in favorable climatic conditions, seeds do not have time to develop or juvenile plants die from the influence of climatic stress. Therefore, the vegetative (clonal) type of reproduction dominates in the populations and the habitat is reduced, which can lead to decreased plant survival in the populations. The previous studies demonstrate that the habitat altitude of the populations has a more significant effect on the productivity indicators of generative organs (number of generative shoots, number of flowers in the inflorescence, mass of formed bulbs, number of bulbs in the seats). Thus, the lowest indicators were observed in the high-mountain populations, average values were observed in low-mountain populations, and high values were observed in the populations located at 1,000–2,000 m above sea level. It is possible that the more favorable conditions in the middle part of the mountains contribute to the appearance of various morphotypes in *A. altaicum* with different adaptive capacity. The middle part has an abundance of vegetation, which provides litter for the subsequent humification of soil cover and ensures its moisture and also favorably affects pollinating insects. These emerging genetic changes in plants can be fixed during clonal reproduction and subsequently ensure the existence of populations in extreme habitat conditions.

Analyzing the indicators that determine the genetic diversity of 12 populations of *A. altaicum*, depending on the ecogeographic area and the altitude of habitat (Table S1; Table 5), it may be said that substantially all meanings are at one level, except for the Shannon biodiversity and the heterozygosity values. Average values of these indicators were higher during the analysis of multiple-altitude groups of populations, rather than during the study of the ecogeographic groups of populations. We suppose that the habitat altitude of a population has a more significant effect on polymorphism than the geographical location of the population. The observed lowering of heterozygosity of populations in the process of ecogeographic division is indicative of the low plant survival rate (possibly as a result of reduced genetic diversity).

It is possible that the distribution area of *A. altaicum* as a relic of the Ice Age was much broader. However, under the influence of climate change and anthropogenic impact (to a large extent, mass collection by the population), groups were fragmented into isolated remote places. Therefore, these populations have close genetic similarities, resulting in a low level of diversity. In addition, relics are characterized by a slow rate of evolution and therefore they are narrowly adapted to certain specific existence conditions.

Heterozygosity indicators were also low in analogous population studies of endemic species of *Allium regelianum* conducted using ISSR analysis. The expected proportion of heterozygous genotypes for all ISSR loci was 0.192; this was higher than in our studies where this indicator was 0.145.

In our studies, analysis of DNA profiling showed that the genetic diversity of *Allium altaicum* populations is due to both interpopulation and intrapopulation differences. Moreover, the genetic diversity within populations depended on their ecological-geographic location. This also indicates that these populations previously shared a common gene pool but were separated by significant isolation barriers. Despite the rather significant genetic differences between all populations, phenotypic changes were not observed with the corresponding geographic distance. For a long period, due to dispersal of seeds by the wind, the inaccessible territories with different climatic conditions were settled, which led to accumulation of intrapopulation polymorphisms. This was shown indicated by the insular or sporadic nature of population dispersal, as sometimes the density is 3–15 individuals per 10 m^2^. The results obtained are consistent with the data; high genetic diversity is promoted by large size and density of populations, while a significant number of individuals can prevent inbreeding and genetic drift (Trifonova, Kochieva & Kudryavtsev, 2017). In addition, the dominant mode of reproduction can also significantly influence genetic diversity. The influence of environmental factors, depending on the gradient of altitude, determines the dominance of vegetative reproduction in populations growing in high mountains (severe stress conditions due to solar radiation, significant temperature amplitude throughout the day) in comparison with populations from medium- and low-altitude regions. In this case, the effect of stress leads to a deficiency of heterozygous genotypes. Based on our research, to develop a strategy for further preserving the gene pool of the relict species *A. altaicum*, it is possible to identify the most genetically distant and variable populations for conservation in a seed bank and several of the most diverse populations that reflect the genetic diversity of the species in a given territory.

## Conclusions

We analyzed 12 populations of various species of the *A. altaicum* species collected in the territory of mountainous and low-land Kazakhstani Altai. Molecular genetic polymorphisms were studied using the PCR method based on PBS primers informative for the genetic analysis of studied samples. This study revealed that the Kazakh populations of *A. altaicum* are distinguished by a high level of genetic diversity. We observed that the degree of genetic differentiation mostly depends on the altitude of habitat rather than the ecogeographic location of the population. Using the iPBS amplification method may solve the problem of species identification of rare endangered species of *Allium* and the preservation of their biological diversity. The *A. altaicum* onion samples from the most distant populations can be used for the preservation and reproduction of the gene pool of this valuable plant species. Our research led to a better understanding of the genetic diversity of natural populations of the *A. altaicum* relict species in the territory of the Kazakhstani Altai. Our findings may contribute to the development of a restoration strategy for the endangered relict *A. altaicum* in the wild.

## Supplemental Information

10.7717/peerj.10674/supp-1 Supplemental Information 1 Primary data for population samples from a different geographical location and analysis using GenAlex 6.5.

10.7717/peerj.10674/supp-2 Supplemental Information 2 Primary data for population samples from a different altitude above sea and analysis using GenAlex 6.5.

10.7717/peerj.10674/supp-3 Supplemental Information 3 Characteristics of the *A. altaicum* samples collected in the territory of the Kazakhstani Altai.

## Abbreviations

AFLP: Amplified fragment length polymorphism
iPBS: Inter-primer binding site
IRAP: Inter-retrotransposon amplified polymorphism
ISSR: Inter simple sequence repeats
LTR: Long terminal repeat
PBS: Primer binding site
RAPD: Random amplification of polymorphic DNA
REMAP: REtrotransposon-microsatellite amplified polymorphism
SCoT: Start codon targeted polymorphism
SSAP: Sequence-specific amplification polymorphism
SSR: Simple sequence repeats

## Additional Information and Declarations

### Field Study Permissions

The following information was supplied relating to field study approvals (i.e., approving body and any reference numbers): Field experiments were approved by the Research Council of the Altai Botanical Garden (Ridder, East Kazakhstan region, Kazakhstan) as a part of project number: BR05236334 (2018–2020), of the Science Committee of the Ministry of Education and Science of the Republic of Kazakhstan in the framework of program funding for research.

### DNA Deposition

The following information was supplied regarding the deposition of DNA sequences: Data is available at GenBank: MK049255.

### Data Availability

The following information was supplied regarding data availability: Raw data is available in the Supplemental Files.
