# Supplementary material for: DNA profiling and assessment of genetic diversity of relict species Allium altaicum Pall. on the territory of Altai
Source: PeerJ. 2021 Jan 8;9:e10674. doi: 10.7717/peerj.10674 (PMC7798630; doi:10.7717/peerj.10674)
Supplement: Supplemental Information 3 [file peerj-09-10674-s003.docx]

**Table S1**. Characteristics of the *Allium altaicum* samples collected in the territory of the Kazakhstani Altai

| **ID number** | **Collection place** | **Coordinates** | | **Altitude, m above sea** | **Number of shoots in the seats, pcs.** | | **Shoot height, cm** | **Number of flowers in the inflorescence, pcs.** | **Number of seeds in the seed balls, pcs.** | **Mass of 1000 seeds, g** | **Bulb diameter, cm** | **Bulb weight, g** | **Number of bulbs in the seat, pcs.** |
| --- | --- | --- | --- | --- | --- | --- | --- | --- | --- | --- | --- | --- | --- |
|  |  | **Latitude** | **Longitude** |  | **vegetative** | **generative** |  |  |  |  |  |  |  |
| Al1 | South Altai Tarbagatai range, Burkhat passage | 49°07'33" | 86°02'10" | 2146 | 0-9 | 1-5 | 59±1.1 | 67.87±4.7 | 2.1±0.4 | 1.85±0,1 | 1.4±0,2 | 4.3±0,5 | 1-5 |
| Al2 | Kalba Range (Eastern Kalba), Koktau Mountains | 49°29'37" | 82°36ʼ19" | 693 | 2-9 | 1-9 | 51±2.0 | 101±4.8 | 4.2±0.3 | 1.10±0.1 | 2.2±0.1 | 16.1±0.9 | 1-7 |
| Al8 | Kalba Range, Taldy Ecosite, Koktau Mountain, North-west slope | 49º29'11' | 82º35'37" | 707 | 1-3 | 4-7 | 57.7±2.0 | 121.8±2.5 | 5.4±0.8 | 1.07±0,1 | 2.1±0,1 | 14.1±0,4 | 3-8 |
| Al9 | Ubinskiy Range, South-eastern slope, Porozhnaya Mountain | 50º33'33" | 82º32'46" | 1800 | 2-4 | 5-8 | 57.7±2.1 | 79.6±8.2 | 5.4±0.8 | 1.15±0.6 | 3.5±0.7 | 25.1±0,7 | 4-9 |
| Al10 | South Altai Tarbagatai, north-west slope, in the area of the Burkhat passage | 50°33'32" | 81°39'30" | 786 | 1-2 | 3-5 | 64.2±0.9 | 59.8±3.9 | 4.2±0.3 | 1.6±0.4 | 3.1±0.1 | 17.9±0.2 | 2-5 |
| Al11 | Narymskiy Range, Kedroviy Kliuch Ecosite, south-west slope | 49º54'34" | 83º44'21" | 1200 | 2-4 | 7-11 | 64.3±2.7 | 104.6±2,4 | 5.2±0.2 | 1.7±0.12 | 2.9±0.5 | 15.6±0.4 | 5-9 |
| Al12 | Site of natural flora of the Altai Botanical Garden | 50º19'34' | 83º32'46" | 774 | 2-7 | 7-11 | 93.3±2.7 | 117.5±3.6 | 5.6±0.4 | 1.85±0.5 | 3.2±0.3 | 19.9±0.2 | 9-19 |
| Al13 | Sarym-Sakty Range, south-east rubble slope, Burkhat | 49º07'33" | 86º02'10" | 2146 | 1-5 | 1-5 | 57±1.4 | 62.87±3.4 | 4.1±0.4 | 1.45±0.1 | 1.4±0.2 | 3.3±0.44 | 1-5 |
| Al14 | Ivanovskiy Range, Seriy Lug Ecosite, north-west slope | 50º19'16" | 83º52'51" | 1800 | 1-4 | 5-9 | 84.2±2.8 | 86.8±3.2 | 5.4±0.6 | 1.45±0.3 | 4.2±0.41 | 19.1±0.5 | 4-9 |
| Al15 | Kalbinskii Altai, Sibin Lakes vicinities. | 49°24'5" | 82°58'23" | 987 | 1-4 | 5-8 | 75.6±2.8 | 112.6±2.7 | 5.1±0.2 | 1.6±0.1 | 2.4±0.7 | 10.6±0.6 | 3-6 |
| Al16 | Western Listvyaga Range, south-east slope, valley of the Kondrashka River | 49°23'16" | 85°45'18" | 1585 | 2-4 | 5-8 | 67.7±2.4 | 81.6±6.2 | 5.2±0.5 | 1.24±0.4 | 3.5±0.7 | 22.1±0.8 | 4-8 |
| Al17 | Kalba Range, Taldy Ecosite, Koktau Mountain, south-east slope | 49º29'18' | 82º35'44" | 915 | 1-4 | 5-8 | 53.7±1.1 | 117.2±2.1 | 5.4±0.2 | 1.23±0.1 | 2.2±0.11 | 12.2±0.4 | 5-8 |
